# 3D-bioprinting for joint regeneration

**DOI:** 10.3389/fbioe.2026.1742269

**Published:** 2026-04-08

**Authors:** Weida Li, Yi Wang, Yue Cui, Qiang Wu, Pu Ying, Kerong Dai, Ye Sun

**Affiliations:** 1 Department of Orthopaedics, The First Affiliated Hospital of Nanjing Medical University, Nanjing, Jiangsu, China; 2 Shanghai Key Laboratory of Orthopaedic Implants, Department of Orthopaedic Surgery, Shanghai Ninth People’s Hospital, Shanghai Jiao Tong University School of Medicine, Shanghai, China; 3 Clinical and Translational Research Center for 3D Printing Technology, Shanghai Ninth People’s Hospital, Shanghai Jiao Tong University School of Medicine, Shanghai, China; 4 Department of Orthopaedics, Changshu Hospital Affiliated to Nanjing University of Chinese Medicine, Changshu, China

**Keywords:** 3D bioprinting, additive components, biomaterials, cells, regeneration

## Abstract

Joint injuries represent a significant clinical challenge with limited regenerative options. Three-dimensional (3D) bioprinting has emerged as a transformative technology, enabling the precise fabrication of patient-specific, anatomically matched, multilayered scaffolds that replicate the complex structure and gradient of natural joint tissues. This review comprehensively summarizes advances in bioprinting techniques, cell sources, and biomaterial formulations, emphasizing cell-laden bioinks composed of biomaterials and viable cells to create functional, bioactive constructs. Beyond basic fabrication, we evaluate the functional performance of bioprinted cartilage, bone, and ligaments, and we discuss strategies for engineering osteochondral interfaces and ligament–bone interfaces to support biomimetic mechanical properties and tissue integration. We further compare major printing modalities, including extrusion-based printing, inkjet, and laser-assisted bioprinting, and we discuss how modality-specific trade-offs in resolution, viscosity window, and cell stress influence construct fidelity and repair outcomes. In addition, we examine biofunctionalization strategies that incorporate growth factors, stem cells, and exosomes to enhance regenerative signaling and matrix remodeling. Notably, 3D bioprinting for joint regeneration is transitioning from bench to bedside, and we detail the current landscape of clinical translation, including commercialized products like Nanochon and active clinical trials for knee and hip repair. However, challenges such as insufficient vascularization and the mechanical performance of the printed constructions remain significant hurdles for clinical translation. Overall, this work underscores the potential of personalized 3D bioprinted scaffolds to advance joint tissue engineering and clarifies key directions for integrating these technologies into clinical practice.

## 1 Introduction

Joint damage profoundly impairs the everyday routines and overall wellbeing of affected individuals, occurring with considerable frequency. Joint injury diseases include not only degenerative osteoarthritis (OA) (Sun et al., 2021a), but also focal joint defects caused by sports injuries. Articular cartilage degeneration is the most prominent feature of osteoarthritis, accompanied by ligament rupture, subchondral osteosclerosis and edema. Articular cartilage is famous for its weak ability to repair due to its lack of vascular supply. Ligaments consist of robust bundles of connective tissue that bridge across a joint, connecting to bones at both ends to ensure joint stability and support its motion. The subchondral bone connects bone and cartilage, which is composed of the upper cortical endplate and the trabecular structure of the lower cancellous bone. At present, there is a lack of active and effective treatment for joint injury, which can only postpone the progress of the defect. In serious cases, joint replacement surgery is needed. However, the artificial prosthesis is a foreign body to the human body and has no activity. The long-term infection and prosthesis loosening limit the curative effect of joint replacement. Although allografts and xenografts serve as feasible substitutes, they are nevertheless plagued by challenges such as immune incompatibility, the potential for rejection, and the spread of infectious pathogens.

Recent advancements in tissue engineering enable the creation of tissues *via* scaffolds that are later populated with cells and supplementary factors. However, the uneven distribution of cells and the lack of blood vessels make the tissue lose its function once implanted in the body. The emergence of three-dimensional (3D) bioprinting provides a remedy for the challenge of uneven cell dispersion in scaffolds, allowing for the fabrication of structures featuring consistent cell arrangement (Subramanian et al., 2024). Meanwhile, a variety of methods to establish vascular system in scaffold have been proposed. Unfortunately, most natural materials used for 3D bioprinting only last for 2–3 weeks before collapsing. On the other hand, artificial substances may exhibit superior mechanical characteristics and can be precisely customized to align with the specific attributes of a particular structure. Nevertheless, artificial substances do not promote cellular proliferation as effectively as their natural counterparts.3D bioprinting can print both synthetic materials used to provide mechanical strength and natural materials used for cell loading simultaneously, thereby seamlessly integrating the characteristics of artificial and organic substances (Buss et al., 2022). Moreover, the process of 3D bioprinting can realize cell-cell interactions and establish signal transduction between cells and the surrounding environment with regulated porosity, pore diameter and scaffold shape. Therefore, the multilayer bionic joint scaffold constructed with 3D bioprinting technology can simulate the structure and gradient of natural joint components (Sun et al., 2021b; Sun et al., 2021c; Sun et al., 2020b) to better realize the regeneration and repair of joints (Mandrycky et al., 2016).

**FIGURE 1:**
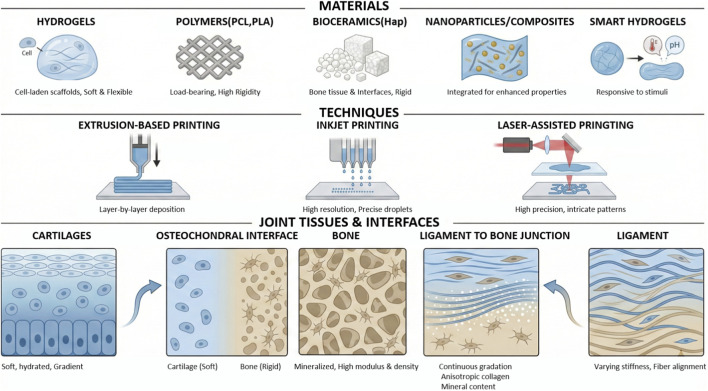
The schematic illustration of the materials and techniques used for the bioprinting of joint tissues and their interface.

This article aims to offer a comprehensive summary of the present-day uses of 3D bioprinting for repairing joints. 3D bioprinting technology, cell types, biomaterials and additive factors selection for joint repair will be discussed below (Figure 1).

## 2 3D bioprinting in joint regeneration

### 2.1 Category of 3D-biopring technology

3D bioprinting is the process of constructing an active structure with cellular bioink (primarily made up of biocompatible substances, viable cells, and/or functional biochemical agents).

#### 2.1.1 Extruded bioprinting

**FIGURE 2:**
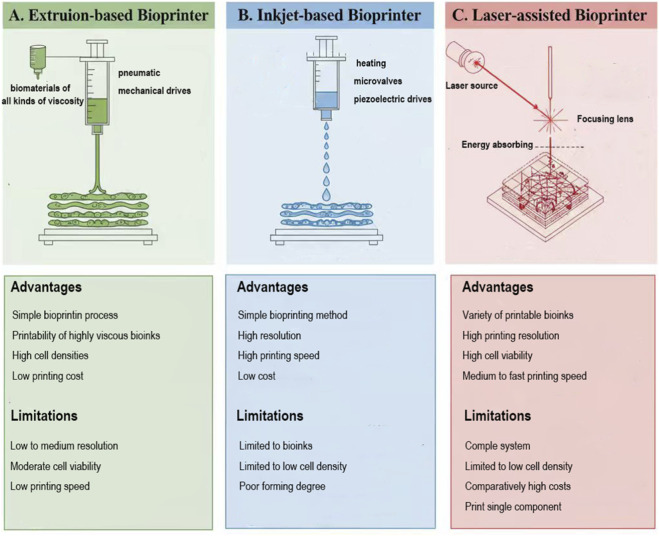
The comparison of three types of bioprinting techniques. **(A)** Extrusion-based Bioprinter: use pneumatic or mechanical drive system as pressure source to extrude biological ink to form the printing structure. **(B)** Inkjet-based Bioprinter: the bioink carrying cells is printed in the form of liquid droplets by means of heating, micro-valve or piezoelectric drive. **(C)** Laser-assisted bioprinting is a technique for the precise patterning of biomaterials, based on the principle of a focused laser energy beam.

Extrusion-based bioprinting: employing a pneumatic or piston-driven mechanism to generate pressure, which allows for the steady dispensing of filament-like bioink, building up successive layers to create a stratified construct. The advantages of extruded bioprinters are:(i) it can print biomaterials of all kinds of viscous property, such as hydrogels, compatible polymers from biological sources, and cellular aggregates with viscosities spanning from 30 to 6 × 10^7^ mPa/s; (ii) modulate and direct the proliferation and specialization of stem cells; (iii) it is cost-effectiveness, accessibility and ability to replicate complex tissue (Figure 2). A few analysts have utilized extrusion-based strategies to bioprint human tissues. Kang et al. fabricated a three-dimensional hydrogel scaffold carrying human amniotic fluid stem cells (hAFSCs) by extruding bioprinting technology and further produced a stable structure of the mandible and skull (Kang et al., 2016). However, numerous limitations, for example, restricted filament precision (typically higher than 100 m) and wrapping of cells subjected to increased pressure in the bioprinting process, restrict the technology’s use and development.

#### 2.1.2 Inkjet bioprinting

Inkjet bioprinting: bioink-carrying cells are printed in the form of liquid droplets by means of heating, microvalves or piezoelectric drives. Inkjet bioprinting offers benefits such as superior precision, elevated processing speed, affordability, consistency, and a notably high cell viability (>90%). (Roth et al., 2004). In addition, inkjet bioprinting can be used to construct multicellular and multicomponent heterostructures, and thermal inkjet bioprinting technology can be used to prepare artificial cartilage. The group used this technique to repair bone and cartilage defects, and the compression modulus of artificial tissue reached 0.4 MPa (Cui et al., 2012a; Cui et al., 2012b). Wu printed a fibrous tissue scaffold using an improved inkjet bioprinter, which highly simulated the layered structure of the tendon from the fiber bundle to the fascicular layer (Wu et al., 2015). Notwithstanding, one significant downside of inkjet bioprinting is the material of decision. The bioink should be fluid and have the proper viscosity to be shot out from the ostiole of the nozzle. The printed bracket exhibits inadequate mechanical strength, while its suboptimal forming quality restricts the broader adoption of this technology.

#### 2.1.3 Laser-assisted bioprinting

Laser-assisted bioprinting: it is a technique for accurate pattern printing of biomaterials based on the principle of laser energy beams (Datta et al., 2018). In laser-assisted bioprinting, the lack of direct contact between the bioink and the dispenser helps prevent contamination and enhances process reliability, a high cell survival rate (>95%) can be obtained. At the same time, laser-assisted bioprinting has high resolution and can accurately print complex structures. In addition, laser-assisted bioprinting is suitable for a wide range of viscosities (1–300 mPa/s) and many kinds of bioinks. Therefore, laser-assisted bioprinting emerges as a promising 3D printing technology for constructing human tissues *in vitro*. Zhu W. et al. (2018) developed a bioprinter based on desktop stereolithography for cartilage biological manufacturing. The sequelae show that the printing resolution and compression modulus of scaffolds can be expressively improved by adding polyethylene glycol diacrylate to the GelMA hydrogel. Catros et al. discovered that laser-assisted 3D bioprinting enables the creation of on-demand patterns using nanohydroxyapatite (HAP) and human osteoprogenitor cells (HOPs) while preserving the survival, proliferation, and phenotype of HOPs while without altering the physicochemical capabilities of nHA (Catros et al., 2011). Although laser-assisted bioprinting offers numerous advantages, the impacts of laser irradiation on cells remain insufficiently investigated. Another barrier to the widespread implementation of this technique is the high equipment costs and the intricate design of the laser printing control system. Additionally, printed scaffolds are typically composed of single materials, which hinders the fabrication of multicellular heterostructures featuring varied compositions and components.

### 2.2 Available cellular opportunities for 3D bioprinting

Cells serve as an important part of tissue regeneration, and cell selection plays a virtual role in engaging in continuous tissue regeneration (Table 1). Depending on the specific requirements for tissue regeneration, a variety of cell subtypes have been employed in 3D bioprinting (Lv et al., 2020; Bernhard et al., 2016; Huang et al., 2016). Pluripotent stem cells, chondrocytes, and human epiphyseal cartilage progenitor cells are the most promising candidates for TE application (Vinatier and Guicheux, 2016).

**TABLE 1:** Cell selection for joint tissue regeneration.

Cells		Tissues differentiation	Advantages	Limitations	References
Pluripotent stem cells	ESC	Bone, cartilage	• Multi-directional differentiation potential	Ethical issues	Craft et al. (2013), Cheng et al. (2014)
	IPSCs	Bone, cartilage	• Pluripotency and self-renewal • No related ethical issues	High cost	Okita et al. (2007), Burke et al. (2016)
	MSC	Bone, cartilage, ligament	• Immunosuppressive properties • Easy to obtain	Limited quantity from relative sources and poor proliferative ability	Burke et al. (2016), Eto et al. (2018), Ma et al. (2009), Tan and Hung (2017)
Chondrocytes		Cartilage	• natural first choice	Small quantity and poor proliferative ability	Lee et al. (2000), Niemeyer et al. (2016), Yin et al. (2018), Choi et al. (2016a), Smeriglio et al. (2015)
hECPs		Cartilage	• High proliferation rates • Stable cartilage differentiation ability	Ethical issues	Darwiche et al. (2012), Broguiere et al. (2016), Carluccio et al. (2020), Studer et al. (2017)

#### 2.2.1 Pluripotent stem cells

The promotion of 3D bioprinting technology has been limited due to insufficient cell sources (Lo et al., 2018). Because pluripotent stem cells possess the capacity to proliferate and differentiate nearly indefinitely into a diverse array of cell types, including osteoblasts, chondrocytes and fibroblasts, they may be a means to overcome this difficulty (Guzzo and O’Sullivan, 2016; Diederichs et al., 2019; Diekman et al., 2012; Yamane et al., 2013; Table 1). Embryonic stem cells (ESCs) refer to pluripotent stem cells that are derived from the inner cell mass of embryos at an early developmental stage. Using extruded-based bioprinting, Ouyang et al. successfully printed scaffolds containing ESC cells, with a good cell survival rate. Some studies have also successfully induced ESCs to differentiate into chondrocytes for joint reconstruction (Craft et al., 2013; Cheng et al., 2014). Nevertheless, ethical considerations and the risk of tumorigenicity continue to pose significant obstacles that need to be addressed prior to their broader application in clinical environments. Induced pluripotent stem cells (iPSCs), which demonstrate pluripotency and self-renewal properties equivalent to those of ESCs, are free from such ethical issues. Recent *in vitro* investigations involving iPSCs derived from diverse cellular sources have demonstrated that these cells exhibit superior proliferation rates and substantial potential for cartilage formation. (Tsumaki et al., 2015; Okita et al., 2007). Nguyen et al. coprinted IPSCs with nanofiber cellulose (NFC). Pluripotency was originally maintained in the 3D bioprinted NFC with alginate (60/40, dry weight percent ratio) constructions, and after 5 weeks, hyaline-like cartilaginous tissue with collagen type II expression was found. IPSCs have a lot of promise for cartilage regeneration, however, the substantial costs involved in the collection, reprogramming, proliferation, differentiation, and transplantation processes remain a significant challenge, as well as their biological danger, making them difficult to deploy. Adult stem cells, like mesenchymal stem cells (MSCs), avoid the ethical and biosafety issues associated with the previously mentioned cells, making them the most widely used source of cells. Because of their immunosuppressive qualities and ability to develop into chondrocytes and osteoblasts, MSCs have been extensively investigated. MSCs can also be isolated from various human tissues, including bone marrow, adipose tissue, synovium, umbilical cord blood, and periosteum (Burke et al., 2016; Eto et al., 2018). Ma et al. (2009) utilized MSCs to create bone-ligament-bone constructs by integrating cell-engineered bone with ligament monolayers. Huang et al. used extrusion-based bioprinting to create a gelatin/hydroxyapatite (HAP) scaffold that was loaded with MSC and effectively stimulated MSC to differentiate into chondrocytes. The scaffold was inserted into the wounded location of porcine articular cartilage and successfully healed the defect. Human bone marrow mesenchymal stem cells (HBMSCs) are the most often employed source of mesenchymal stem cells in therapeutic applications (Tan and Hung, 2017). HBMSCs can be readily isolated from donors and differentiate into chondrocytes for hyaline cartilage regeneration and osteoblasts for joint tissue regeneration. Similar to the clinical treatment of microfractures, HBMSCs can be mobilized to differentiate into chondrocytes or osteoblasts for tissue regeneration.

#### 2.2.2 Chondrocytes

Mature chondrocytes from articular cartilage represent the natural first choice, commonly used in laboratory studies, clinical trials, and therapies (Table 1). Typically, articular chondrocytes are isolated from small tissue biopsies obtained from healthy, presumably non-load-bearing regions. While this methodology may yield a high-quality cellular source, it does so at the expense of a healthy cartilage region, putting patients at risk for OA (Lee et al., 2000). Furthermore, the bioactivity of isolated chondrocytes declines with age, making older individuals unsuitable as chondrocyte donors (Niemeyer et al., 2016). In addition, the insufficient size of biopsy specimens and the low density of chondrocytes mean that the chondrocytes that can be isolated are far from sufficient for clinical use. The prevailing solution entails cultivating cells *in vitro*; nevertheless, chondrocytes propagated in two-dimensional monolayer cultures relinquish their characteristic spherical morphology, acquire fibroblast-like attributes, and undergo dedifferentiation, thereby forfeiting numerous qualities that render them suitable for bone and cartilage regeneration (Yin et al., 2018). Additional investigations have looked into other chondrocyte sources. Chondrocytes at immature developmental stages, encompassing fetal, infantile, and juvenile variants, have a better potential to proliferate and create cartilage than mature chondrocytes, according to P Smeriglio (Choi WH. et al., 2016; Smeriglio et al., 2015), suggesting that they might be a therapeutically viable source of allogeneic cells. To date, scant reports have documented the incorporation of articular chondrocytes into microgels, primarily owing to the constrained availability of this cell type; nevertheless, one investigation involved culturing articular chondrocytes on particles sourced from the cartilage extracellular matrix, enabling the formation of functional cartilage-like microtissues characterized by elevated expressions of cartilage-specific markers.

#### 2.2.3 Human epiphyseal chondroprogenitors

Human epiphyseal cartilage progenitor cells (hECPs) are a novel source of progenitor cells produced from the donor’s proximal ulnar epiphysis during 14 weeks of gestation (Darwiche et al., 2012). It is expected tissue differentiation capabilities and stability make them a promising cartilage regeneration option (Broguiere et al., 2016). In addition, hECP has a high proliferation rate, which makes the yield of a single tissue donor very high, so the resulting cell population has better homogeneity than the source of mature cells (Darwiche et al., 2012; Carluccio et al., 2020; Table 1). Nevertheless, it is essential to underscore that research involving this cell type mandates preliminary ethical authorization, which may delay entry of these cells into clinical trials. Nevertheless, owing to their substantial long-term potential, an expanding volume of research is dedicated to the utilization of these fetal cell types in articular cartilage regeneration (Broguiere et al., 2016; Studer et al., 2017). Although HECPs are promising candidates for cartilage regeneration, there is little evidence to assess their effectiveness in 3D bioprinting. More research is needed to see how well it performs in 3D bioprinting.

For joint reconstruction, a variety of cells have been tried. Presently, induced pluripotent stem cells represent the most prevalently utilized cell type. However, the future direction may be to use a variety of cells at the same time, differentiate into corresponding tissue structures, and jointly participate in the reconstruction of complex structures.

#### 2.2.4 Cell co-culture systems for joint tissue regeneration

To better recapitulate the complex physiological microenvironment of native joints, various cell co-culture systems have superseded single cell type approaches in 3D bioprinting (Deszcz and Bar, 2025). Recent studies emphasize the synergy between MSCs and chondrocytes in enhancing cartilage repair. For instance, Apelgren et al. successfully generated cartilage-like structures *in vivo* using a 1:4 ratio of chondrocytes to MSCs in alginate hydrogels (Apelgren et al., 2017). Similarly, Critchley et al. utilized 3D-printed polycaprolactone scaffolds and alginate gel to co-culture subpatellar adipose MSCs and chondrocytes at a 3:1 ratio, achieving superior repair in goat osteochondral defect models (Critchley et al., 2020). By optimizing these co-culture systems, 3D bioprinting technology can more effectively mimic the cellular interactions and regenerative potential of natural joint tissues, ultimately leading to more successful tissue engineering strategies for joint regeneration.

### 2.3 Biomaterials in 3D bioprinting

3D bioscaffolds are composed of scaffolding structures and cell carriers. The ideal scaffolding structure met the following requirements: (i) Biocompatibility. It is nontoxic and does not cause immunogenic reaction or inflammatory reactions. (ii) Biodegradability. Regulated scaffold degradation can facilitate tissue ingrowth while preserving adequate structural integrity. (iii) Bioactivity. Scaffold materials capable of interacting with and adhering to the host tissue. (iv) Scaffold architecture. Diffusion and cell migration are facilitated by interconnected pores. (v) Reliable mechanical properties. To date, numerous natural and synthetic biomaterials have undergone evaluation for joint tissue regeneration both *in vitro* and *in vivo* (Turnbull et al., 2018; Semba et al., 2020; Table 2). Other techniques have been considered, such as the utilization of decellularized cartilage ECM (Critchley and Kelly, 2017) and bioactive glasses.

**TABLE 2:** Biomaterials for joint tissue regeneration.

Biomaterials		Tissues reconstruction	Representative function	References
Natural carbohydrate-based polymers	Sodium alginate	Cartilage	• Promote MSC differentiate • Maintain chondrocytes phenotype	Almeida et al. (2016), Wang et al. (2011), Witte et al. (2020)
	Chitosan	Cartilage	• Enhanced cell adhesion and proliferation	Khatab et al. (2020), Kang et al. (2014), Zhou et al. (2016)
	Hyaluronic acid	Cartilage	• Promote chondrocyte adhesion, proliferation	Erickson et al. (2021), Feng et al. (2020)
Protein-based polymers	Collagen	Cartilage	• Stimulate cell proliferation and differentiation	Ti et al. (2016), Sawadkar et al. (2019), Mozdzen et al. (2016)
	Gelatin	Bone, cartilage	• Supporting cell adhesion, migration and proliferation	Feng et al. (2019), Li et al. (2020), Van den St et al. (2002)
Synthetic polymers	PCL	Bone, ligament	• Slow degradation rate • Less acidic breakdown products	Kim and Kim (2015), T et al. (2017)
	PLA	Bone, ligament	• Biodegradable • Bioactive thermoplastic polyester	Holmes et al. (2016), Cooper et al. (2005)
	PEG	Cartilage, bone, ligament	• Non-toxic • Low pollution • Highly soluble in water and organic solvents	Zhu and Marchant (2011), Qiu et al. (2011), Ma et al. (2020), Sharma et al. (2013), Paxton et al. (2009), Woodruff and Hutmacher (2010), Gloria et al. (2010)

#### 2.3.1 Natural carbohydrate-based polymers

Sodium alginate can facilitate the differentiation of bone marrow-derived mesenchymal stem cells into chondrocytes while preserving their phenotypic characteristics (Almeida et al., 2016; Wang et al., 2011; Witte et al., 2020; Table 2), which makes it one of the preferred materials for joint regeneration. Chitosan is a kind of polysaccharide that can be degraded by enzymes *in vivo* (through lysosomes) (Khatab et al., 2020). And the structure of chitosan is similar to that of glycosaminoglycan, consequently, it demonstrates augmented cellular adhesion and proliferation, thereby sustaining the chondrogenic phenotype and facilitating the deposition of cartilaginous extracellular matrix (Kang et al., 2014; Zhou et al., 2016; Table 2). Hyaluronic acid, alternatively termed hyaluronan (HA), represents a principal constituent of articular cartilage. It works in tandem with agglutinin to give an elastic structure and facilitate the condensation process (Erickson et al., 2021; Feng et al., 2020). Furthermore, HA is capable of engaging with multiple cell surface proteins, such as CD44, ICAM-1, and RHAMM, thereby enhancing chondrocyte adhesion, proliferation, and metabolic processes (Table 2). For bone regeneration, Nath mixed HA and chitosan in porous scaffolds and found stem cell colonization and proliferation in the scaffold (Nath et al., 2014). Antich et colleagues found that co-printed scaffolds made of HA and polylactic acid can promote chondrocyte development and maintenance while also providing the necessary mechanical qualities. However, there are several drawbacks to using natural polymers, such as the exice of pathogenic contaminants like endotoxin, the inability to tune degradation rates, and local cellular inhibition caused by breakdown. Natural polymers exhibit inferior mechanical properties relative to ceramics and metals.

#### 2.3.2 Protein-based polymers

Protein-based biomaterials mainly use collagen (Ti et al., 2016) or gelatin (Feng et al., 2019; Li et al., 2020) as biomolecules.

Collagen constitutes a principal component of the intra-articular extracellular matrix and exhibits negligible immunoreactivity. As a constituent of the standard extracellular matrix, it exhibits inherent biocompatibility and biodegradability, while also promoting cellular proliferation and differentiation (Table 2). The obtained tendons showed similar mechanical strength and no adhesion formation to the native tendons (Sawadkar et al., 2019). Employing 3D bioprinting techniques, Lee and colleagues developed a collagen-based silk-fibroin scaffold with ECM-like properties and a nanoporous architecture. *In vitro* evaluations demonstrated that the fabricated construct provided an exceptionally supportive milieu for osteoblast differentiation, attributable to its densely integrated ECM-mimetic attributes. Nevertheless, relative to human cancellous bone tissue, the construct displayed significantly diminished mechanical strength and compressive modulus (Mozdzen et al., 2016). Gelatin preserves several collagen’s features, such as low immunogenicity. Furthermore, gelatin demonstrates biodegradability, with potential degradation facilitated by matrix metalloproteinases (MMPs), such as MMP-2 and MMP-9 (Van den St et al., 2002). Gelatin has a high biocompatibility, allowing cells to adhere, migrate, and reproduce, as well as stimulate bone and cartilage formation (Yue et al., 2015; Table 2). Kim et al. employed a bioink composed of gelatin and a poly (vinyl alcohol) (PVA) solution to fabricate scaffolds mimicking hard tissue, intended for bone regeneration under low-temperature conditions. Incorporating PVA into the gelatin scaffold enhanced its mechanical strength. To improve bioprintability while keeping the porous structure, several gelatin/PV A composition ratios were tried. Finally, a 5:5 scaffold ratio was shown to be the most favorable for high cell viability and osteogenic differentiation.

#### 2.3.3 Synthetic polymers

Synthetic polymers utilized in joint tissue engineering encompass polycaprolactone (PCL), polylactic acid (PLA), and polyethylene glycol (PEG). PCL has been extensively applied in the fabrication of three-dimensional scaffolds for joint tissue engineering. Its advantages include biocompatibility, a relatively slow degradation rate, the production of less acidic degradation byproducts compared to other polyesters, and suitability for load-bearing applications. Kim et al. demonstrated the efficacy of PCL/alginate composite scaffolds, reporting improved osteoblast cell viability, calcium deposition, alkaline phosphatase (ALP) activity, and cell seeding efficiency (Kim and Kim, 2015). Shi et al. demonstrated that a biomimetic ligament fabricated with PCL and nanohydroxyapatite had excellent mechanical properties comparable to those of human rotator tendons (Tong et al., 2017). It shows that PCL is an ideal material for joint tissue regeneration (Table 2).

Polylactic acid (PLA) constitutes a biodegradable and bioactive thermoplastic polyester that has been employed in the fabrication of bone screws and scaffolds. Holmes et al. fabricated polylactic acid (PLA)-based scaffolds employing fused deposition modeling 3D printers. Not only can the synthetic scaffold promote bone formation and angiogenesis, but it can also endure typical mechanical loads and exhibit elastic behavior (Holmes et al., 2016; Table 2). Cooper et al. (2005) described a multiphased synthetic ACL graft made from three-dimensional braiding of polylactide-coglycolide fibers that included a ligament proper as well as two bone regions. Although few research on the use of 3D bioprinting to rebuild joint ligaments exist. However, this endeavor shows that 3D bioprinting ligaments are feasible, and more study is needed to assess the mechanical qualities and biofriendliness of printed ligaments.

Because it is nontoxic, low polluting, easily soluble in water and organic solvents, and has been authorized by the FDA for numerous biomedical uses, PEG is the most extensively utilized synthetic polymer in joint tissue engineering applications (Zhu and Marchant, 2011; Table 2). PEG generally binds to RGD peptides, fibronectin, laminin, or collagen to improve cell adhesion (Qiu et al., 2011; Ma et al., 2020). Gao et al. employed inkjet bioprinting to fabricate three-dimensional bone constructs through the co-deposition of acrylated peptides and acrylated polyethylene glycol (PEG) alongside marrow-derived human mesenchymal stem cells (MSCs) (Lo et al., 2018). Following differentiation, the bioprinted bone niche exhibited substantial deposition of calcified mineralized matrix, coupled with encouraging mechanical properties essential for bone architecture. Moreover, the mechanical stiffness and intricate architecture of the bioprinted hydrogels demonstrated a direct induction of mesenchymal stem cells (MSCs) toward osteogenic lineages. (Lo et al., 2018). Investigations have revealed that photo-encapsulated bone marrow-derived mesenchymal stem cells (MSCs) within PEGDA hydrogels exhibit cartilage formation and matrix deposition (Sharma et al., 2013). Paxton et al. (2009) successfully designed the attachment of ligaments to bone by adding HA and RGD peptides to PEG hydrogels at the same time. However, these are still preclinical studies, and more studies are needed to explore the feasibility of applying this technology to clinical practice in order to accelerate clinical transformation. In general, the load-bearing capacity of synthetic and natural polymers is relatively poor when used alone, and the elastic modulus is lower than that of metal and ceramic compounds (Woodruff and Hutmacher, 2010). As a result, combining polymers with bioceramics or bioglass to create composite scaffolds could be a solution to this problem. In most circumstances, adding bioceramic or bioglass to the polymer matrix as a coating or filler can increase the scaffold’s biological activity as well as its mechanical qualities (Gloria et al., 2010).

### 2.4 Additive component selection

To maintain cell growth and differentiation, additive components are mixed into biomaterials with cells. Growth factors, exosomes, and microRNAs are all examples (Table 3). Numerous growth factors have been incorporated into the domain of joint regeneration (Craft et al., 2013; Sun et al., 2019). The most extensively utilized growth factors and hormones that encourage stem cell differentiation are transforming growth factor (TGF), insulin-like growth factor (IGF) (Okita et al., 2007), bone morphogenetic protein (BMP) (Zhang et al., 2017), vascular endothelial growth factor (VEGF) (Ben-David et al., 2013), and parathyroid hormone (PTH) (Liang et al., 2018). The TGF family of proteins plays a key role in embryonic development, tissue morphogenesis, cellular proliferation, and differentiation (Table 2). Research has demonstrated that TGF-β1 and TGF-β3 can facilitate the differentiation of bone marrow mesenchymal stem cells into cartilage, preserve the chondrocyte phenotype, and ultimately contribute to the regeneration of cartilage and osteochondral tissue (Lo et al., 2018; Table 3). The BMPs, particularly BMP-2, BMP-4, and BMP-7, represent the most extensively studied osteogenic factors for inducing *de novo* bone formation in both ectopic and orthotopic sites, including critical-sized defects (Burke et al., 2016; Table 3). VEGF and IGF are capable of modulating angiogenesis while facilitating bone formation. The possible pathway is that VEGF acts on vascular endothelial cells to produce bone morphogenetic protein (BMP) and other substances, which act as osteogenic signals to interact with osteoblasts (Eto et al., 2018). Although the methods by which PTH directs osteogenic activity are unknown, investigations have demonstrated that PTH may increase bone growth in rats and humans (Diederichs and Tuan, 2014; Table 3). Exosomes originate from the exocytosis of living cells and contain cargo inside. Studies have shown that exosomes can enhance the formation and integration of joint tissue (Bei et al., 2021). Exosomes, according to Zhang et al., can stimulate cell proliferation and infiltration, promote matrix protein synthesis, and have anti-inflammatory properties (Zhang et al., 2018; Table 3). The microRNAs(miRNAs) secreted by osteoblasts represent a new coupling factor released by osteoblasts (Xu et al., 2021; Zhao et al., 2021). A series of miRNAs regulating bone formation of osteoblasts have been identified and divided into two types: osteoblast-derived miRNAs and osteoblast precursor-derived miRNAs (Zhu S. et al., 2018; Table 3). Cui et al. found that miRNA-677-3β enhances MSC osteogenic differentiation by regulating axis inhibitory protein 1 (Cui et al., 2016).

**TABLE 3:** Additive bioactive for cellular differentiation and regeneration.

Additive components		Tissues treated	Representative function	Ref
Growth factor	TGF-β1	Bone	• Proliferation and differentiation of bone-forming cells	Iwakura et al. (2013)
	TGF-β3	Cartilage	• Enhances cartilage formation *in vivo*	Lo et al. (2018)
	BMP-2	Bone	• Differentiation and migration of osteoblasts • Enhanced bone healing and increased bone mechanical strength	Du et al. (2015)
	BMP-4	Bone		Burke et al. (2016)
	BMP-7	Cartilage	• Promote the proliferation and differentiation of chondrocytes	Iwakura et al. (2013)
	BMP-10	Cartilage		Zhang et al. (2008)
	VEGF	Bone	• Promote osteogenesis	(Zhang et al., 2012), (Schmitt et al., 2013)
	IGF	Bone; vessel	• Enhanced vasculogenesis • Enhance proliferation and differentiation of osteoprogenitor cells	Eto et al. (2018)
	PTH	Bone	• Accelerated bone formation	Diederichs and Tuan (2014)
MSC-derived exosomes		Cartilage	• Encourage the growth of chondrocytes	(Thomas et al., 2021), (Jiang et al., 2021)
Osteoclast-derived exosome		Bone	• Enhance the formation and integration of bone	(Bei et al., 2021), (Zhang et al., 2018)
Myoblasts-derived exosome		Ligament	• Enhance muscle regeneration and differentiation	Choi et al. (2016b)
miRNA-677-3β		Bone	• Enhances MSC osteogenic differentiation	(Zhu et al., 2018b), (Cui et al., 2016)
Scleraxis		Ligament	• Elevated tendon gene expression • Attenuated adipogenic potential	Font et al. (2015)

Although numerous growth factors have been employed to enhance cellular proliferation and differentiation, its potential cancer-promoting effect still warns us that we need to use it with caution. Controlling the release of growth factors is also an urgent problem to be solved.

### 2.5 Cell carrier

Although the aforementioned biomaterials have been employed as scaffold substrates and have enough mass to offer the support and qualities required for tissue development, their cell simulation quality and contact with stromal cells are poor, which is critical for tissue regeneration (Mantha et al., 2019; Naghieh et al., 2019). Another way to overcome the limitations of these polymer scaffolds is to design cell carriers, for example, print structures based on hydrogels (Caló and Khutoryanskiy, 2015; Sun et al., 2020a). Hydrogels are gels made up of crosslinked, hydrophilic polymer chains that form a network. Because of their hydrophilic nature, they are capable of absorbing substantial quantities of water within a three-dimensional network, rendering them highly suitable for cellular development (Moukbil et al., 2020). As the composition and structure of hydrogels closely resemble those of the extracellular matrix (ECM) in various human tissues, hydrogels are easy to prepare under relatively mild and hydrochemical conditions and are considered to be a suitable environment for cell development in scaffolds (Chen, 2019; Chung et al., 2013). The elevated water content in these materials establishes a hydrated tissue environment that is optimal for cell integration and enhances cell viability during bioprinting within a hydrated and mechanically stable three-dimensional framework (Fu et al., 2017). The structural characteristics of hydrogels can increase the permeability of oxygen nutrition and the excretion of metabolic waste, which attributes supports the proliferation and differentiation of cells within the scaffold. Scaffolds constructed from hydrogels have been extensively utilized in the field of tissue engineering owing to their exceptional adaptability in aspects such as size, shape, composition, and related properties. Angiogenesis, osteoconductivity, cell adhesion, and matrix integration have all been demonstrated to be aided by hydrogels.

### 2.6 Smart responsive biomaterials

Smart responsive biomaterials are designed to undergo reversible or programmable changes when exposed to external cues or *in vivo* microenvironmental stimuli. These triggers may involve variations in temperature and pH, enzymatic activity or reactive oxygen species, as well as optical, magnetic, electric, and mechanical inputs. In response, the materials can exhibit swelling or shrinkage, gelation, stiffness switching, shape morphing, controlled degradation, and on-demand drug release (Raei et al., 2025; Wu et al., 2010).

In joint regeneration, particularly for cartilage repair and osteochondral interface reconstruction, their responsiveness to lesion-associated biochemical conditions and mechanical loading supports spatiotemporally regulated biochemical cues and mechanical microenvironments, which can enhance cell survival, promote chondrogenic differentiation, stimulate extracellular matrix deposition, and improve integration with subchondral bone (Xu et al., 2025; Yang et al., 2020). Endogenously responsive smart biomaterials exploit the joint-injury/osteoarthritis microenvironment to achieve self-triggered regulation of regeneration. During articular cartilage injury and osteoarthritis, the local milieu is commonly characterized by elevated oxidative stress with increased reactive oxygen species, acidification with decreased pH, and aberrant upregulation of matrix metalloproteinases such as MMP-13 (Liu et al., 2010; Xiang et al., 2024). These pathological features provide intrinsic stimulus sources for endogenously triggered smart responsive materials, enabling lesion-site–specific adaptive degradation, on-demand drug release, and remodeling of the regenerative microenvironment (Qin et al., 2026). Unlike endogenously triggered responses, physically exogenous responsive materials emphasize non-invasive or minimally invasive control through external fields such as magnetic fields, light, or ultrasound. These materials are characterized by switchable and tunable regulation, making them especially suitable for integration with 3D bioprinting to develop 4D bioprinting (Chakraborty et al., 2024; Yang H. et al., 2025).

A digital light processing based three-dimensional bioprinting strategy has been reported in which three photocrosslinkable, cell laden bioinks were employed to enable covalent interlayer coupling and the incorporation of a mineralization gradient, resulting in near complete repair of osteochondral defects in a rat model (Yang X. et al., 2025). Liu et al. developed a composite scaffold consisting of piezoelectric poly lactic acid and strontium silicate ceramic and further applied clinically used low intensity pulsed ultrasound as an exogenous trigger to facilitate integrated osteochondral regeneration (Liu et al., 2025). Xie et al. designed a piezoelectric three-dimensional composite scaffold and introduced collagen coating together with fibroblast growth factor 18 delivery in the bottom layer, thereby generating a synergistic interplay between piezoelectric stimulation and bioactive factor signaling and ultimately enhancing cartilage repair (Xie et al., 2024).

### 2.7 Functional performance of bioprinted tissues

#### 2.7.1 Bone

In recent years, 3D bioprinting technology has been widely utilized for the production of bone tissue substitutes, with its developmental trajectory evolving from mere structural biomimicry toward sophisticated functional emulation. Wang et al. (2023) utilized fused deposition modeling (FDM) to produce large-scale bone scaffolds by integrating polylactic acid (PLA) with nano-sized β-tricalcium phosphate (β-TCP) at a 7:3 ratio. These scaffolds featured customizable porosity and geometric configurations, achieving complete healing within 12 weeks in a rabbit femoral defect model, with osteogenic activity markedly surpassing that of pure PLA controls. Addressing the challenge of fabricating unsupported, overhanging ceramic structures in a single printing step, Raja et al. (2023) introduced a “support-free ceramic printing” (SLCP) strategy. This approach utilized a thermoresponsive Pluronic P123 hydrogel bath as a reversible support medium, enabling one-step fabrication of complex anatomies such as the mandible. The enhanced surface roughness of the scaffolds resulted in a twofold increase in cellular adhesion capacity and a marked upregulation in the expression of osteogenesis-related proteins. Li T. et al. (2023) leveraged selective laser sintering (SLS) to integrate modified polyvinyl alcohol (MPVA) with hydroxyapatite (HAP), producing porous “bone bricks” with 68.3% porosity and interconnected channels. *In vivo* assays revealed pronounced upregulation of alkaline phosphatase (ALP) and Runx2 gene expression, alongside superior new bone formation relative to controls. Guerrero et al. (2025) incorporated osteoblast-derived mineralized exosomes into 3D-printed HA/TCP ceramic gyroid scaffolds. In a rabbit calvarial defect model, this design achieved osteoinduction without exogenous growth factors, significantly enhancing osteogenic gene expression and accelerating mineralization, thereby establishing a “cell-free, intrinsically bioactive” platform. Kim et al. (2024) engineered blade-stacked microstructures (LSS) on polycaprolactone (PCL) scaffold filaments and immobilized bone morphogenetic protein-2 (BMP-2) to realize sustained release over 32 days. This synergistic design promoted cell adhesion, proliferation, and differentiation, expediting bone regeneration markedly compared to conventional smooth filament scaffolds. Brito et al. (2025) developed a portable extrusion device capable of *in situ* printing composite filaments composed of PLA, nanoclay, and hydroxyapatite at ambient temperature, directly applied to rat cranial defects. Rapid cooling circumvented thermal damage, facilitating continuous bone bridging within 8 weeks without fibrous capsule formation—heralding a novel paradigm for “on-demand, personalized” bone repair. Collectively, these advancements underscore how integrative innovations in materials, architecture, and bioactivity within 3D bioprinting are propelling bone tissue engineering toward expedited clinical translation.

#### 2.7.2 Cartilage

Advances in 3D bioprinting in the field of cartilage tissue engineering may be concisely categorized into four primary areas. Firstly, in printing technologies, Wu Y. et al. (2024) pioneered embedded printing techniques that directly deposit low-viscosity bioinks into supportive baths, achieving high-fidelity stacking of micron-scale arch networks, thereby effectively mitigating structural collapse and recoil. Complementarily, Reineke et al. (2024) synergized microfluidics with 3D printing to continuously generate homogeneous cell-laden microgels within microfluidic chips, subsequently assembling them *via* a “flow-to-print” modality into multicellular, multiscale fibrous constructs—offering a novel platform for rapid fabrication of complex cartilage architectures.

Secondly, with respect to cellular strategies, Hu et al. (2023) harnessed human umbilical Wharton’s jelly to fabricate photocrosslinkable AWJMA-GelMA hydrogels encapsulating only second-passage bone marrow stromal cells (BMSCs), achieving over 95% viability at 7 days. The intrinsic TGF-β-rich microenvironment of the umbilical matrix facilitated *in situ* chondrogenic induction. Similarly, Pei et al. (2023) validated in a rabbit full-thickness knee cartilage defect model that GelMA-MSC scaffolds restored collagen II and glycosaminoglycan levels near physiological benchmarks after 12 weeks, substantiating a “low-passage, rapid implantation” paradigm that expedites clinical translation.

Thirdly, innovations in bioink formulations include Zengin et al. (2023) incorporation of mesoporous silica nanoparticles functionalized with matrix metalloproteinase (MMP)-sensitive peptides into a degradable norbornene-based hydrogel, enabling precise matching of cartilage region-specific moduli ranging from 9.3 to 19.7 kPa. This system synchronizes degradation and tissue remodeling upon cell-secreted MMP-9 activation. Kansız et al. (2025) engineered methacrylated κ-carrageenan/hydroxyapatite composite bioinks by modulating κ-carrageenan substitution degrees, endowing the ink with a yield stress of 450 Pa and immediate post-extrusion shape fidelity. The hydroxyapatite nanocrystals confer bidirectional osteochondral bioactivity, facilitating integrated bone-cartilage interface printing.

Lastly, regarding scaffold architecture, Chakraborty et al. (2023) employed dual bioinks comprising GelMA and silk fibroin-gelatin to recapitulate, *in vitro*, the 50–80 μm arched collagen networks characteristic of articular cartilage. This biomimicry elevated compressive modulus to 350 kPa and, following 21 days of BMSC culture, quadrupled Col2A1 and SOX9 expression. Díaz-Payno et al. (2023) demonstrated a high/low swelling bilayer printing approach to achieve liquid-triggered four-dimensional (4D) bending, transforming planar constructs into auricular-shaped cartilage with a 4 mm radius of curvature within 6 hours. This morphology was stably maintained with sustained glycosaminoglycan content over 28 days, exemplifying a clinically viable “one-step printing—self-morphing” strategy for curved cartilage such as ear, nose, and trachea.

Looking forward, the field necessitates the development of more intelligent biomimetic bioinks, integration of growth factor and co-culture systems, and refinement of printing technologies to enhance resolution and the capacity to fabricate intricate architectures. These advances will collectively propel the standardization and maturation of cartilage organoid research.

#### 2.7.3 Ligament

Over the past few years, 3D bioprinting has experienced significant progress in the field of ligament tissue engineering, particularly demonstrating immense potential in recapitulating the intricate structural and functional complexity of ligaments. Given the pronounced anisotropic architecture inherent to tendon and ligament tissues, conventional bioprinting approaches have struggled to achieve precise cellular alignment, thereby constraining enhancements in mechanical integrity and biomimicry (Kim and Kim, 2023). Addressing this challenge, Kim D and colleagues devised a hybrid printing strategy combining high-concentration acellular bioinks as supportive matrices with shear flow-induced cellular orientation, successfully fabricating a composite tendon construct characterized by a rigid exterior and an integrated interior. This approach substantially enhanced the tenogenic differentiation of human adipose-derived stem cells (hASCs) (Kim and Kim, 2023).

From a materials science standpoint, tissue-specific decellularized extracellular matrix (dECM) bioinks have achieved broad implementation due to their exceptional biocompatibility and intrinsic tissue-inductive capabilities in the development of cell-laden ligament constructs. Chae et al. pioneered an integrated “3D printing plus *in vitro* pre-maturation” strategy, wherein stem cells encapsulated within tissue-specific dECM were induced *in vitro* to form semi-organized tendon-like tissues. Subsequent implantation into nude mice markedly enhanced cell viability and mechanical strength of the neo-tendon (Chae et al., 2022). To further bolster the mechanical stability and functionality of printed architectures, Weng H et al. developed a methacrylated collagen oligopeptide-xanthan gum (COPMA-XG) interpenetrating network hydrogel. This material not only exhibited favorable printability and self-healing properties but also sustainedly promoted human mesenchymal stem cells (hMSCs) differentiation toward ligament phenotypes (Weng et al., 2025).

In pursuit of higher-order biomimetic structures, four-dimensional (4D) printing has been introduced to fabricate molecularly aligned collagen scaffolds. By fine-tuning the collagen-to-xanthan gum ratios and incorporating magnetically responsive microparticles, Patrawalla NY et al. achieved highly oriented collagen molecular arrangements without compromising printing fidelity, effectively steering stem cell differentiation toward tendon/ligament lineages (Patrawalla et al., 2024). Additionally, the integration of near-infrared (NIR) photopolymerization with upconversion nanoparticles (UCNPs) has unveiled new avenues for *in vivo in situ* printing. This technique enables direct fabrication of personalized ligament scaffolds within the body, minimizing surgical trauma and enhancing tissue integration efficiency, thereby charting a promising course for minimally invasive therapies (Xiao et al., 2025).

Despite these significant breakthroughs, insufficient vascularization remains a critical bottleneck impeding functional maturation and long-term viability of bioprinted ligament constructs. To surmount this, Chae S and colleagues proposed co-printing endothelial cells or incorporating pro-angiogenic factors to engineer functional vascular networks, thereby elevating physiological relevance and tissue integration capacity (Chae et al., 2023a).

In summary, 3D bioprinting technology manifests expansive prospects within ligament tissue engineering. Through concerted innovations in biomaterials, structural design, and integrative methodologies, it is poised to realize biomimetic ligament constructs endowed with pronounced cellular orientation, mechanical congruence, vascularization potential, and seamless interface integration—offering transformative solutions for clinical ligament injury repair.

#### 2.7.4 Osteochondral and ligament-bone interfaces

The osteochondral interface and ligament-bone junction serve as biological transition zones between different tissues, are characterized by complex structural and functional gradients that are essential for physiological stress distribution and mechanical stability (Fan et al., 2021; Fang et al., 2024). These regions are highly susceptible to injury due to their hierarchical heterogeneity (Benjamin et al., 2006).

**FIGURE 3:**
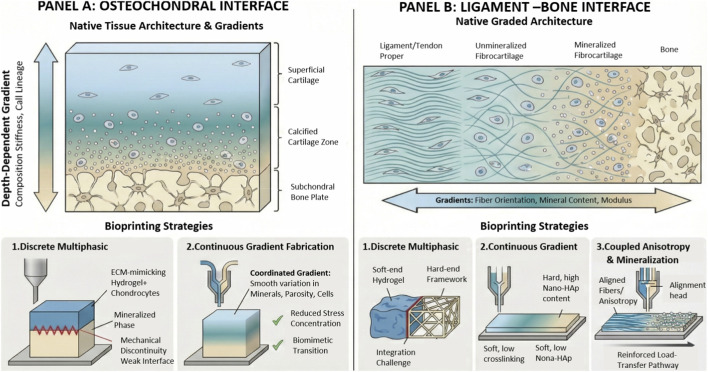
The schematic illustration of the osteochondral and ligament-bone interfaces.

Osteochondral tissue exhibits pronounced depth-dependent gradients in composition, cellular lineage, and mechanical properties (Ansari et al., 2019). The superficial cartilage layer is highly hydrated and low-modulus, and it transitions downward into the highly mineralized, high-modulus regions of calcified cartilage and the subchondral bone plate (Mansfield et al., 2015). Therefore, the key to gradient 3D bioprinting lies in simultaneously reconstructing a coordinated and continuous transition across biochemical, cellular, structural, and mechanical dimensions (Abaci and Guvendiren, 2024). Existing strategies can be broadly classified into two categories (Figure 3). The first is discrete layered or multiphasic construction, in which multiple printheads or hybrid printheads switch between or co-deposit different bioinks during printing (Ege and Hasirci, 2023). In the cartilage region, ECM-mimicking hydrogels are typically used and loaded with chondrocytes or MSCs to induce chondrogenic differentiation, whereas in the bone region, a mineralized phase (hydroxyapatite or bioglass) is incorporated to increase stiffness and promote osteogenic differentiation (Lu et al., 2025). This approach is process-stable and straightforward to implement, but it may suffer from mechanical discontinuities and insufficient integration at the interlayer interfaces (Suo et al., 2021). The second approach is continuous gradient fabrication, which uses in-line proportional mixing or microfluidic printheads, high-resolution photopolymerization techniques, and spatially graded distributions of nanoparticles or microspheres to create continuous variations in mineral content, porosity and microarchitecture, cell density, and growth-factor release kinetics (Davoodi et al., 2020; Wang et al., 2022; Dormer et al., 2010). These coordinated gradients can reduce interfacial stress concentrations and more closely recapitulate the transitional microenvironment of native osteochondral units (Xie et al., 2025; Liu et al., 2024). However, while more biomimetic, this strategy imposes stricter requirements on printing resolution, material compatibility, and gradient stability, and it still demands engineering trade-offs among structural fidelity, cell viability, and long-term mechanical performance (Jung et al., 2025).

The ligament-to-bone interface is a representative soft-to-hard tissue transition with a continuous gradation in structure and function. This transition is commonly organized into distinct yet continuous regions, including the ligament/tendon proper, unmineralized fibrocartilage, mineralized fibrocartilage, and bone (Liu et al., 2024; Xu et al., 2024). This graded architecture is accompanied by coupled variations in collagen fiber orientation and tissue anisotropy, mineral content, and mechanical modulus. Consequently, the objective of gradient 3D bioprinting is not merely to assemble multiple materials within one construct, but to establish coordinated gradients in structure, composition that promote region-specific differentiation and reduce interfacial stress peaks (Chae et al., 2023b).

Existing fabrication strategies can be broadly grouped into three categories. (i) Discrete multiphasic printing employs a mechanically robust “hard-end” framework printed from thermoplastic polymers or ceramic-based composites such as polycaprolactone combined with calcium phosphate, followed by deposition of cell-laden hydrogels to form a compliant “soft-end” ligament phase (Cao et al., 2020). The intermediate transition layer is often incorporated to improve interfacial integration and load transfer. This route is generally process-stable and offers tunable mechanical properties, yet interlayer continuity and long-term integration remain major challenges (Chen et al., 2020; Bai et al., 2025). (ii) Continuous gradient printing uses in-line proportional mixing, microfluidic mixing modules, or coaxial core–shell printheads to generate spatially continuous changes in parameters such as nanohydroxyapatite content, crosslinking density, porosity, cell density, and the release kinetics of growth factors including TGF-β and BMP family cues (Xie et al., 2025; Kim et al., 2025). While this approach more closely recapitulates the native graded microenvironment and can mitigate interfacial stress concentrations, it requires high gradient fidelity, rheological compatibility among materials, and sustained gradient stability in the presence of diffusion, particle sedimentation, and differences in crosslinking kinetics. (iii) Coupling anisotropy gradients with mineralization gradients aims to reinforce the load-transfer pathway across the insertion site (Sensini et al., 2021). Alignment on the ligament side can be introduced *via* printing-induced shear, guidance microgrooves, or fiber-reinforcement strategies including short-fiber incorporation, textile-based architectures, or hybrid electrospinning–printing constructs, while mineral content and stiffness are progressively increased toward the bone side (Uehlin et al., 2022). This integrated design concept is increasingly viewed as a key direction for enhancing functional regeneration of the enthesis and lowering the risk of re-rupture.

#### 2.7.5 Vascularization

Joint tissues have significant regional vascularization needs. Articular cartilage typically maintains a low or avascular state, while subchondral bone, the osteochondral interface, and the peripheral regions of ligaments and menisci rely on blood supply for nutrient exchange, mineralization, and integration (Huang et al., 2024). Therefore, the goal of vascularization in joint bioprinting is not to globally increase vascularity, but to construct a perfusable vascular network with spatially controlled distribution, while preventing abnormal vessel invasion into the cartilage region that could lead to degeneration risks (Santos-Beato et al., 2022).

In bioprinted osteochondral and joint-related constructs, vascularization strategies are commonly grouped into two overarching paradigms, direct vascular bioprinting and indirect inductive vascularization. Direct vascular bioprinting introduces vascular features during fabrication to create predefined transport pathways. Representative implementations include coaxial extrusion to generate hollow lumens, sacrificial inks that are removed after printing to yield perfusable channels, and embedded or freeform printing approaches, sometimes combined with microfluidic elements, to build interconnected channel networks (Kjar et al., 2021; Liu et al., 2022). By alleviating diffusion constraints, these engineered conduits can improve cell survival in thicker tissues and provide a spatial template that supports endothelialization and may facilitate post-implantation anastomosis (Budharaju et al., 2024). Indirect vascularization, in contrast, does not necessarily require preformed continuous lumens but promotes angiogenesis and vascular stabilization through biological induction. This paradigm encompasses controlled delivery of pro-angiogenic factors such as VEGF, including synergistic or temporally staged presentation with maturation-associated cues, co-printing of endothelial cells with supportive stromal populations such as MSCs or pericyte-like cells and or *in vitro* prevascularization to enable microvascular self-assembly, and architectural design features such as porosity and spatial gradients in material composition or mechanics that encourage host vessel ingrowth and remodeling (Rofaani et al., 2024; Revokatova et al., 2025; Kawai, 2025). Overall, indirect strategies emphasize microenvironmental instruction and self-organization together with host-driven infiltration to establish functional vascular networks.

**FIGURE 4:**
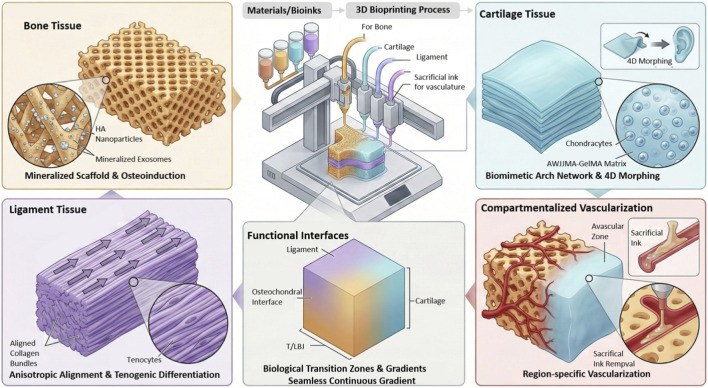
Functional performance of bioprinted tissues.

A key requirement for joint tissue engineering is compartmentalized vascularization. The subchondral bone region and the osteochondral interface benefit from adequate perfusion to support osteogenesis and integration, whereas the cartilage region is physiologically avascular and excessive vascular invasion is typically detrimental to cartilage homeostasis (Hu et al., 2021; Wei and Dai, 2021). Accordingly, zonal design frameworks are increasingly proposed that combine perfusable channels on the bone side as a direct strategy, pro-maturation inductive signaling at the interface as an indirect strategy, and an anti-angiogenic barrier or transitional layer toward the cartilage side to limit vascular penetration (Seymour et al., 2022; Wu D. et al., 2024). This integrated region-specific approach aims to better align engineered constructs with native osteochondral vascular biology while meeting the distinct functional demands of bone, interface, and cartilage compartments (Figure 4).

### 2.8 Organoid

Organoid technology, as an innovative paradigm, has emerged with expansive potential in the realm of articular cartilage repair (Dutta et al., 2017; Rossi et al., 2018). Organoids, which are three-dimensional tissue structures generated from stem cells *in vitro*, accurately replicate the complex architecture and physiological functions of native organs, thus providing highly physiologically relevant models for biomedical research (Dutta et al., 2017; Rossi et al., 2018). Leveraging three-dimensional (3D) bioprinting, articular organoids can precisely emulate the *in vivo* joint microenvironment, encompassing the multifaceted interplay among cartilage, synovium, and bone tissues, thus serving as robust models for drug screening and biomaterial evaluation of implants (Chen S. et al., 2022; Wang et al., 2021; Gao et al., 2017). The quintessential objective in constructing joint organoids lies in generating functional tissue substitutes *ex vivo* to replace damaged cartilage and facilitate regenerative repair. Concurrently, 3D printing technology affords customizable design capabilities, enhancing both the mechanical robustness and biocompatibility of the constructs (Gao et al., 2017; Zhu et al., 2016). This integrative strategy not only accelerates the advancement of personalized medicine but also unveils novel therapeutic avenues for degenerative joint diseases (Li ZA. et al., 2023).

#### 2.8.1 Strategies for constructing articular organoids

The construction of articular organoids can be systematically categorized into three hierarchical tiers: basic organoids, structural organoids, and functional organoids (Xie et al., 2022). Basic organoids primarily emphasize the judicious selection of appropriate cellular sources—such as induced pluripotent stem cells (iPSCs) or mesenchymal stem cells (MSCs)—alongside biomaterials like gelatin methacryloyl (GelMA) or hydroxyapatite (HAP), aiming to faithfully recapitulate the native joint tissue microenvironment. For instance, iPSCs can be differentiated into chondrocytes to fabricate cartilage organoids instrumental for joint repair (Abe et al., 2023). The choice of biomaterials necessitates a balanced consideration of mechanical robustness and biocompatibility to optimally support cellular proliferation and differentiation. Structural organoids use sophisticated 3D printing techniques, including digital light processing (DLP) and extrusion-based methods, to meticulously design scaffold architectures, including gradient porosities and microspheres, thereby emulating the native Haversian system of bone (Xie et al., 2022; Zhang et al., 2020). Such meticulous structural design enhances mechanical integrity while promoting homogeneous cellular distribution. For example, microspheres fabricated *via* DLP printing have demonstrated elevated cell viability and facilitated stem cell differentiation (Xie et al., 2022). Functional organoids incorporate bioactive elements—such as growth factors including transforming growth factor-beta (TGF-β), bone morphogenetic proteins (BMPs), and extracellular vesicles—to regulate vascularization, immunomodulation, and the initiation of signaling pathways, thereby enhancing the physiological performance of the organoids (Chen et al., 2018; Song et al., 2019). The incorporation of vascular endothelial growth factor (VEGF), for instance, stimulates neovascular network formation, while microfluidic technologies enable the recreation of complex multi-tissue interactive milieus (Kelleher et al., 2016). Collectively, these functionalization strategies propel organoids closer to mimicking the dynamic *in vivo* joint microenvironment.

#### 2.8.2 Applications of articular organoids

Three-dimensional (3D) bioprinted articular organoids have been broadly utilized in fields including disease modeling, drug screening, and regenerative medicine. These organoids provide highly physiologically relevant platforms for investigating joint pathologies such as osteoarthritis and rheumatoid arthritis, overcoming the constraints intrinsic to conventional two-dimensional (2D) cultures and animal models (Li ZA. et al., 2023; Yi et al., 2021). By faithfully recapitulating pathological states, articular organoids enable in-depth exploration of inflammatory mechanisms and tissue degeneration processes. In the realm of drug development, articular organoids facilitate high-throughput screening to evaluate anti-inflammatory agents and osteogenic stimulants, as well as for evaluating the biocompatibility of implantable biomaterials, such as metals and ceramics (Makarczyk et al., 2021; Chen Y. et al., 2022). From a personalized medicine perspective, organoids derived from patient-specific cells allow for the customization of therapeutic regimens, thereby enhancing treatment precision and efficacy (Chen Y. et al., 2022). Furthermore, articular organoids contribute to joint repair by engineering cartilage and bone tissues that promote regeneration; for example, by inducing stem cell differentiation into functional phenotypes to replace damaged tissue (Feng et al., 2023; Lawlor et al., 2021). These multifaceted applications accelerate the advancement of joint disease therapies while concurrently reducing the costs associated with clinical translation. Collectively, the construction and deployment of articular organoids through 3D bioprinting technology enable precise emulation of the joint microenvironment, furnishing powerful tools to propel future clinical research endeavors (Lawlor et al., 2021).

## 3 Clinical translation

Overall, 3D bioprinting for joint regeneration is still in a transitional stage, moving from feasibility validation toward clinical translation (Li et al., 2010). Several joint-related personalized implants or scaffold-type medical devices based on 3D bioprinting concepts have already achieved commercialization, including Nanochon Chondrograft, Cytex ReNew Hip Implant, iTotal Identity, and the G7 Acetabular System. In addition, clinical trials involve knee (NCT07312175, NCT01957722, NCT07249489), hip (NCT06823089), and ankle (ChiCTR2500115790). Although personalized scaffold fabrication can currently be realized based on imaging such as CT/MRI, and biomimetic multilayer structures can be built through precise spatial control of cells, materials, and bioactive factors, further improvements are still needed in structural complexity, simulation of spatial heterogeneity, and ultimately clinical applicability (Picado-Tejero et al., 2025).

Key bottlenecks that hinder clinical translation remain prominent. These include insufficient vascularization, which limits the long-term survival and functional maturation of constructions (Li et al., 2010). They also include inadequate mechanical performance and mismatched material degradation kinetics, which compromise structural stability and tissue remodeling in load-bearing joint tissues (Liu et al., 2024). To address these bottlenecks, one strategy is composite bioprinting that combines synthetic and natural materials to balance mechanical support with cell friendliness. Another strategy is using polymer bioceramic or polymer bioglass composites to enhance both mechanical properties and bioactivity. A further strategy is to apply pro-angiogenic induction approaches together with more advanced printing paradigms such as gradient printing and smart responsive materials to improve physiological relevance and translational feasibility (Chen et al., 2024; Elango and Zamora-Ledezma, 2025).

## 4 Conclusion

3D bioprinting technology has introduced a transformative framework for joint regeneration by facilitating the accurate placement of cells, biomaterials, and bioactive agents to construct biomimetic multilayered scaffolds that accurately replicate the inherent structure and functionality of articular joints. This adaptable technology integrates a variety of approaches—such as extrusion-based printing, inkjet printing, and laser-assisted bioprinting—and employs an array of cellular sources, including pluripotent stem cells and chondrocytes, in combination with both natural and synthetic biomaterials, to facilitate the functional restoration of cartilage, ligaments, and bone tissues. The emergence of organoid technology has additionally enhanced the accuracy of simulating the joint microenvironment, thus enabling advanced disease modeling, high-throughput drug screening, and individualized therapeutic strategies. Nonetheless, the clinical translation of 3D bioprinting for joint regeneration remains constrained by insufficient vascularization, inadequate load bearing mechanical integrity, and mismatched biomaterial degradation-tissue remodeling. Progress will require better bioink design, improved construction architecture, and stronger vascularization strategies. Standardized and scalable manufacturing and evaluation pipelines are also needed.

Future trajectories are poised to converge upon hybrid printing strategies—exemplified by 4D bioprinting—integrated smart biomaterials, and angiogenic induction approaches, all aimed at enhancing the physiological relevance and translational viability of engineered constructs. Despite existing hurdles, the synergistic integration of 3D bioprinting and organoid technologies holds immense promise to propel joint regeneration from bench to bedside, ultimately realizing the aspirational goal of personalized, bioengineered tissue repair.

## Glossary

3D: Three-dimensional
4D: Four-dimensional
ACI: Autologous chondrocyte implantation
ACL: Anterior cruciate ligament
ALP: Alkaline phosphatase
BMP: Bone morphogenetic protein
BMSCs: Bone marrow–derived mesenchymal stem cells
CT: Computed tomography
DIW: Direct ink writing
DLP: Digital light processing
ECM: Extracellular matrix
ESCs: Embryonic stem cells
FDM: Fused deposition modeling
FGF18: Fibroblast growth factor 18
HAP: Hydroxyapatite
HBMSCs: Human bone marrow mesenchymal stem cells
HIF: Hypoxia-inducible factor
HOPs: Human osteoprogenitor cells
IGF: Insulin-like growth factor
iPSCs: Induced Pluripotent Stem Cells
LIPUS: Low-intensity pulsed ultrasound
MMPs: Matrix metalloproteinases
MRI: Magnetic resonance imaging
MSCs: Mesenchymal stem cells
NFC: Nanofibrillated cellulose
OA: Osteoarthritis
PCL: Polyglycolide
PEG: polyethylene glycol
PLA: Polylactic acid
PTH: Parathyroid hormone
RA: Rheumatoid arthritis
RGD: Arg–Gly–Asp peptide motif
ROS: Reactive oxygen species
SLS: Selective laser sintering
TCP: Tricalcium phosphate
TGF-β: Transforming growth factor beta
UCNPs: Upconversion nanoparticles
VEGF: Vascular endothelial growth factor
